# Data on flow cell optimization for membrane-based electrokinetic energy conversion

**DOI:** 10.1016/j.dib.2017.08.036

**Published:** 2017-09-01

**Authors:** David Nicolas Østedgaard-Munck, Jacopo Catalano, Mette Birch Kristensen, Anders Bentien

**Affiliations:** Department of Engineering, Aarhus University, Hangoevej 2, 8200 Aarhus N, Denmark

**Keywords:** Electrokinetic energy conversion, Electrochemical flow cell, Conversion efficiency

## Abstract

This article elaborates on the design and optimization of a specialized flow cell for the measurement of direct conversion of pressure into electrical energy (Electrokinetic Energy Conversion, EKEC) which has been presented in Østedgaard-Munck et al. (2017) [1]. Two main flow cell parameters have been monitored and optimized: A) the hydraulic pressure profile on each side of the membrane introduced by pumps recirculating the electrolyte solution through the flow fields and B) the electrical resistance between the current collectors across the combined flow cell. The latter parameter has been measured using four-point Electrochemical Impedance spectroscopy (EIS) for different flow rates and concentrations. The total cell resistance consists of contributions from different components: the membrane $\left(R_{\mathrm{mem}}\right)$, anode charge transfer $\left(R_{A}\right)$, cathode charge transfer $\left(R_{C}\right)$, and ion diffusion in the porous electrodes $\left(R_{D}\right)$.

The intrinsic membrane properties of Nafion 117 has been investigated experimentally in LiI/I_2_ solutions with concentrations ranging between 0.06 and 0.96 M and used to identify the preferred LiI/I_2_ solution concentration. This was achieved by measuring the solution uptake, internal solution concentration and ion exchange capacity. The membrane properties were further used to calculate the transport coefficients and electrokinetic Figure of merit in terms of the Uniform potential and Space charge models. Special attention has been put on the streaming potential coefficient which is an intrinsic property.

**Specifications Table**

**Table:** 

Subject area	*Chemical engineering*
More specific subject area	*Membrane technology for energy conversion*
Type of data	*Tables, graphs, figures*
How data was acquired	Electrochemical Impedance Spectroscopy (CH Instruments, CHI660E) Conductivity probe (eDAQ, platinum plate electrodes, cell constant k = 10 cm^-1^). Autotitrator (Metrohm Autotitrator (916Ti-Touch))
Data format	*Raw, analysed*
Experimental factors	Nafion 117 pretreatment: boiled in 3 wt% H_2_O_2_ for 1 h. Washed in boiling in milli-Q water for 10 min. Boiled in 0.05 M sulfuric acid for 30 min. Washed several times in boiling water. After pretreatment the membrane was stored in 1 M LiCl until use.
Experimental features	*Construction of a specialized flow cell on which 4-point EIS was performed in flow using LiI/I*_*2*_*solutions.* *Solution uptake measured as the mass of: (wet membrane – dry membrane)/dry membrane.* *Internal membrane solution concentration determined by measuring the conductivity related to the amount of excess ions sorbed and later released from the membrane upon immersion in water.* *Ion exchange capacity done by washing a solution soaked piece of membrane in water. Then the membrane was transferred to a hydrochloric acid solution of known concentration. End concentration, determined by titration, then yields the ion exchange capacity*
Data source location	*Hangoevej 2, 8200 Aarhus N, Denmark*
Data accessibility	*Data is displayed within this article*
Related research article	*This Data in Brief article is submitted as a companion paper to:* *Østedgaard-Munck, D. N., Catalano, J., Kristensen, M. B., & Bentien, A. (2017). Membrane-based electrokinetic energy conversion. Materials Today Energy, 5, 118–125.*

**Value of the data**

● Flow cell optimization with respect to flow patterns, electrical resistance, and general cell design.
● Data analysis method and results for Electrochemical Impedance Spectroscopy on operating flow cells.
● Optimization of the solution concentration including measurements of:

a) Solution uptake
b) Internal solution concentration
c) Ion exchange capacity

● Modelling of membrane transport properties using two classical pore models (Uniform potential and Space charge models).

## 1 Data

### 1.1 Flow cell pressure profile

The flow regime in the graphite plate channels (see Fig. 6) was evaluated considering the Reynolds number, Re, which is calculated as:

(1) Re=uρextDHμext−1withDH=4Acxp−1

Assuming: (*i*) 12 channels with equal fluid velocity $u=\frac{1}{12A_{c}}q_{\mathrm{circ}}$ (in m s^-1^) where $q_{\mathrm{circ}}$ is the circulation flow rate (in m^3^ s^-1^) and $A_{c}$ is the cross sectional area (in m^2^), (*ii*) solution density $\rho_{\mathrm{ext}}≅\rho_{w}$ ~ 10^3^ kg m^-3^ and viscosity $\mu_{\mathrm{ext}}≅\mu_{w}$~ 10^-3^ Pa s (assumed equal to that of water at 25 °C). $D_{H}$ = 10^-3^ m is the hydraulic equivalent diameter of a single square channel (width w=1 mm and height h=1 mm) having wetted perimeter $x_{p}$. For the conditions used in the present work Re = 16.7–267 for $q_{\mathrm{circ}}$ = 0.2–3.2 mL s^-1^ which ensures that all experiments were conducted in laminar flow conditions in the flow field channels.

**Fig. 1:**
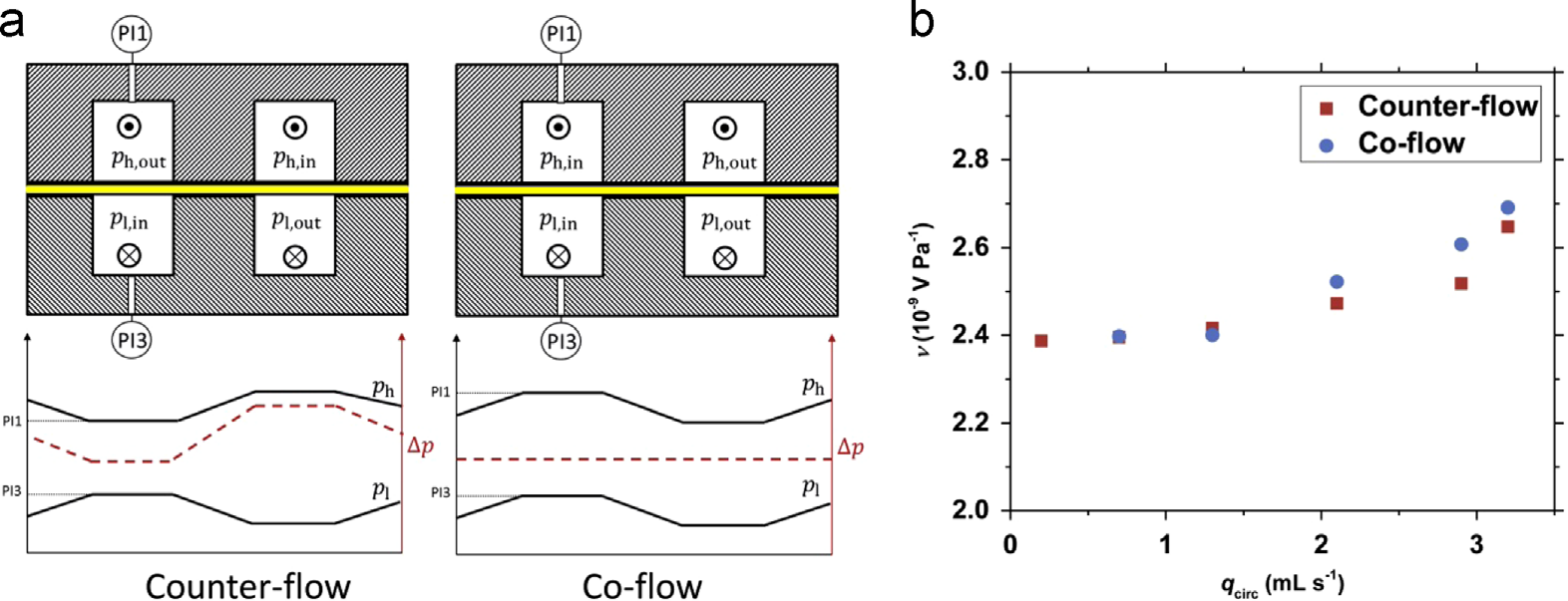
a) Schematic of the counter-flow and co-flow conditions for the fluid flow in the interdigitated channels showing the profiles of the qualitative pressures (solid lines) and transmembrane pressure difference (dashed lines. b) Streaming potential coefficients versus the circulation flow rate in counter and co-flow scaled by the logarithmic mean pressure difference.

The pressure drop in each half cell was measured with two pressure indicators positioned between the internal (inlet) and the outlet port. Streaming potential coefficient measurements (at zero current density) where performed both with co-flow and counter flow conditions (see Fig. 1a) and were used to validate the pressure differences measured in the system. The data can be seen in Fig. 1b scaled with the logarithmic mean pressure difference.

### 1.2 Flow cell electrochemical impedance spectroscopy

**Fig. 2:**
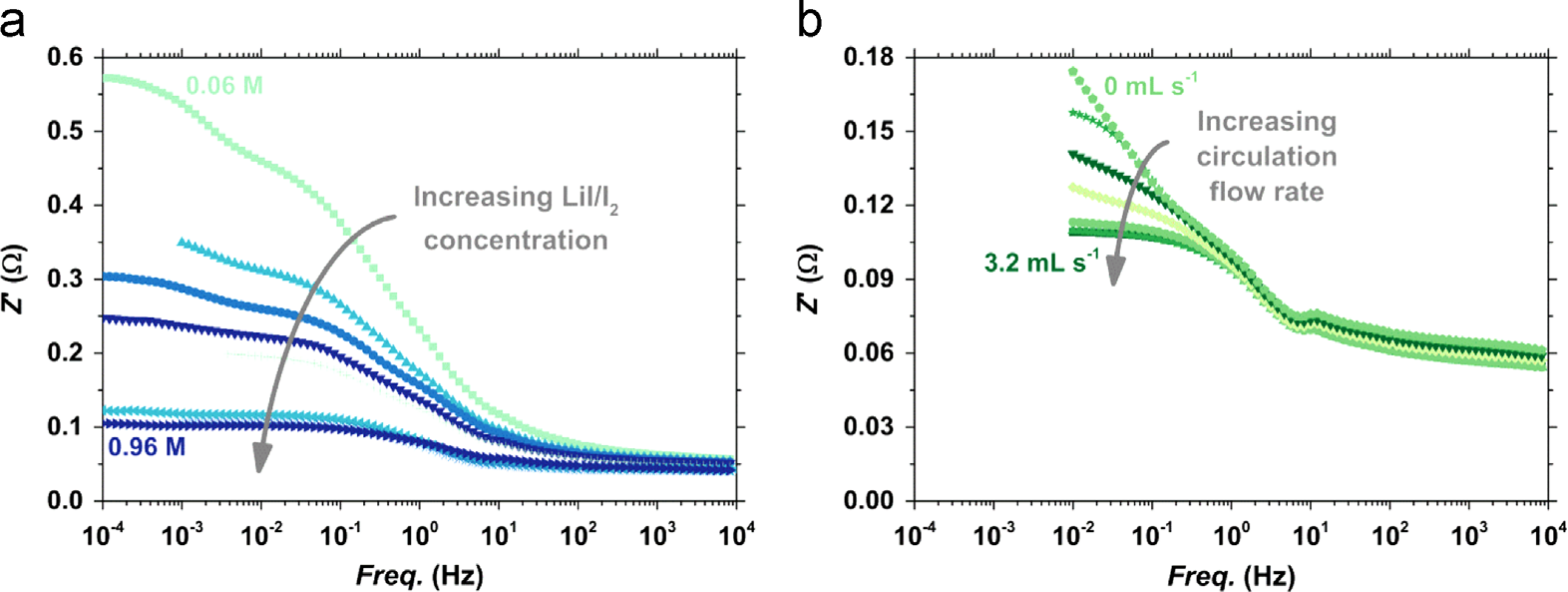
a) Bode plots for varying LiI/I_2_ concentrations (0.06–0.96 M) at fixed *q*_circ_=2.9 mL s^-1^. b) Bode plot for varying circulation flow rates (0–3.2 mL s^-1^) at fixed *c*_ext_=0.26 M LiI/I_2_.

The Bode plots for varying concentrations and circulation flow rates can be seen in Fig. 2a and b, respectively. Corresponding Nyquist plots can be seen in ref [1].

### 1.3 Membrane properties

See Table 1.

**Table 1:** Experimental data for solution uptake (*s*), ion exchange capacity (*iec*), internal solution concentration of LiI $\left({\tilde{c}}_{\exp}\right)$ saturated with I_2_ and immobile charge density (${\tilde{X}}_{t}$), and calculated internal solution concentrations (${\tilde{c}}_{\mathrm{UP},s}$, ${\tilde{c}}_{\mathrm{UP},t}$) for two different immobile charge densities (${\tilde{X}}_{s}$ and ${\tilde{X}}_{t}$).

$c_{\mathrm{ext}}$ (M)	*s* ($g_{\mathrm{sol}}g_{\mathrm{dry pol}}^{-1}$)	*iec* ($\mathrm{meq}g_{\mathrm{pol}}^{-1}$)	${\tilde{X}}_{t}$ (mol m^-3^)	${\tilde{c}}_{\exp}$ (M)	${\tilde{c}}_{\mathrm{UP},s}$^a^ (M)	${\tilde{c}}_{\mathrm{UP},t}$^b^ (M)
0.06	0.20	–	–	0.0028^⁎^	0.00084	0.0011
0.12	0.23	–	–	0.0051^⁎^	0.0034	0.0042
0.16	0.19	–	–	0.0062	0.0060	0.0075
0.21	0.24	–	–	0.012	0.010	0.013
0.26	0.24	0.80	3528	0.017	0.016	0.020
0.51	0.22	0.80	3445	0.045	0.060	0.075
0.74	0.18	0.83	3539	0.062	0.125	0.154
0.96	0.23	0.78	3189	0.144	0.206	0.252
Average	0.21±0.02	0.80±0.02	3425±163	–	–	–

*Notes*: ^⁎^ Conductivity below detection limit. ^a, b^ The immobile charge densities used for the Donnan equilibrium calculations were ${\tilde{X}}_{s}$ = 4270 and ${\tilde{X}}_{t}$ = 3400 mol m^-3^, respectively. The subscripts “s” and “t” indicates whether the ion exchange capacity used in the calculations comes from the manufacturer “specifics” (*iec ~* 0.91 meq $g_{\mathrm{pol}}^{-1}$) or measured with titration (*iec ~* 0.80 meq $g_{\mathrm{pol}}^{-1}$).

### 1.4 Electrical potential profiles and Donnan equilibrium

To have insights on the optimal solution concentration for EKEC processes, the internal solution concentration was determined theoretically considering two classical pore models (Uniform potential and Space charge models). Generally a nanocapillary of radius *R* with charged walls (σ, in C m^-2^) in equilibrium with an external electrolyte solution (with concentration $c_{\mathrm{ext}}$) develops cation and anion concentration profiles along the radial direction (*r*) which can be calculated from the Boltzmann distribution as:

(2) $$c_{i}\left(r\right)=c_{\mathrm{ext}}\exp\left(\;-\;z_{i}\psi \left(r\right)/\Phi_{B}\right)\;[\mathrm{mol}\;m^{-3}]$$

where $z_{i}$ (dimensionless) and $\Phi_{B}$ are the valence of the *i*th ion and the thermal voltage ($\Phi_{B}=R_{g}T/F$ (in V) with *R*_g_, *T* and *F* being the gas constant (in J mol^-1^ K^-1^), absolute temperature (in K) and Faraday constant (in C mol^-1^). Here the ions are modelled as point charges and no effects related to the ion size and deviation on the dielectric constant of the medium are taken in consideration. The electrical potential ψ (in V) can be calculated from the Poisson equation in cylindrical coordinates for a 1:1 electrolyte as:

(3) $$\frac{1}{r}\frac{d}{\mathrm{dr}}\left(r\frac{d\psi \left(r\right)}{\mathrm{dr}}\right)=\frac{F}{\epsilon }c_{\mathrm{ext}}\sinh\left(-\frac{z_{i}\psi \left(r\right)}{\Phi_{B}}\right)$$

where ϵ is the permittivity of the medium (6.9×10^-10^ F m^-1^). Eq. (3) must be solved with the proper boundary conditions which, for the case of interest here, are constant charge density on the pore wall (*r=R*) and symmetry at *r*=0:

(4) $${\frac{d\psi \left(r\right)}{\mathrm{dr}}|}_{r=R}=\frac{\sigma }{\epsilon },{\frac{d\psi \left(r\right)}{\mathrm{dr}}|}_{r=0}=0$$

The solution of the Poisson equation (Eq. 3) is central in the “Space charge” model theory and the calculation of the internal solution concentration (as integral of $c_{i}\left(r\right)$ on the radial direction) will be referred to as “SC” [2], [3], [4] In the limiting case of constant electrical potential profile inside the pore (which is strictly valid when the pore radius is smaller than the characteristic Debye length $\lambda ={\left(\frac{\epsilon \Phi_{B}}{2Fc_{\mathrm{ext}}}\right)}^{0.5}$) the internal solution concentration (${\tilde{c}}_{\mathrm{calc}})$ can be determined as:

(5) $${\tilde{c}}_{\mathrm{calc}}=\frac{1}{2}{\tilde{c}}_{t}\;-\;\frac{1}{2}\tilde{X}=\frac{1}{2}{\left({\tilde{X}}^{2}+\;4c_{\mathrm{ext}}^{2}\right)}^{0.5}-\;\frac{1}{2}\tilde{X}\;[\mathrm{mol}\;m^{-3}]$$

which coincide with the one calculated from Donnan equilibrium [5], [6]. In Eq. (5) ${\tilde{c}}_{t}={\left({\tilde{X}}^{2}+4c_{\mathrm{ext}}^{2}\right)}^{0.5}$ is the total ion concentration inside the membrane. The approximation of constant concentration profile inside the pore (or overlapping electrical double layers) is the main assumption in the “Uniform potential” model and the internal concentration calculated with Eq. (5) will be referred to as “UP” [2], [3], [7], [8]

**Fig. 3:**
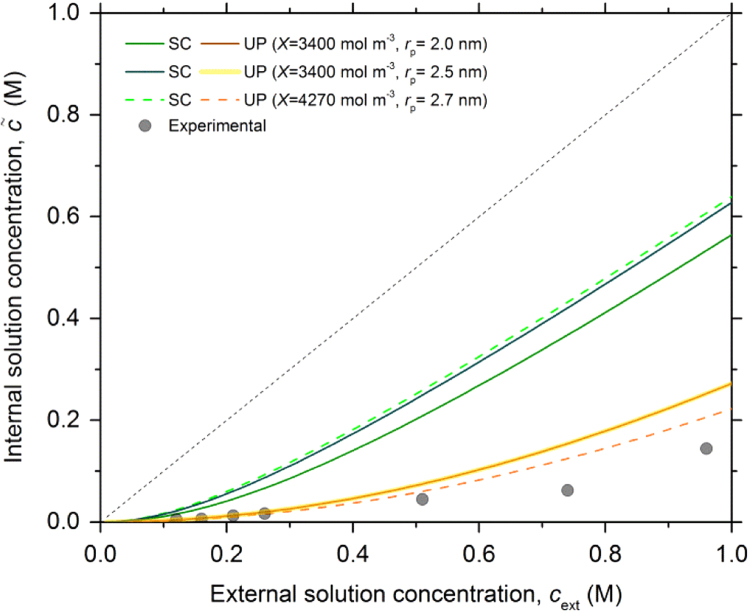
Internal LiI/I_2_ solution concentration ($\tilde{c}$) as function of the external solution concentration ($c_{\mathrm{ext}}$) measured and determined based on two different models; Space charge (SC) (*green curves*) and Uniform potential (UP) (*yellow curves*) with different immobile charge densities ($\tilde{X}$) and pore radii ($r_{p}$). Experimentally determined internal solution concentrations are represented by solid symbols.

In Table 1 ${\tilde{c}}_{\mathrm{calc}}$ determined with ${\tilde{X}}_{s}$ and ${\tilde{X}}_{t}$ are given as ${\tilde{c}}_{\mathrm{UP},s}$ and ${\tilde{c}}_{\mathrm{UP},t}$, respectively. The influence of $c_{\mathrm{ext}}$ on ${\tilde{c}}_{\exp}$ is shown in Fig. 3 together with ${\tilde{c}}_{\mathrm{calc}}$ derived from the SC and UP models (see ref. [4], [8]) with $\tilde{X}$ equal to 3400 and 4270 mol m^-3^ and pore radii of 2.3, 2.5 and 2.7 nm, respectively. The pore radii used in these calculations have been chosen to obtain a good fit with the experimental streaming potential data at infinite dilute conditions (see next section) and are similar to the one measured from SAXS experiments in Nafion [9].

Systematically ${\tilde{c}}_{\exp}$ are lower than ${\tilde{c}}_{\mathrm{UP}}$ calculated from the Donnan equilibrium which means that the effective immobile charge density might be higher than the one estimated from titration (i.e. ${\tilde{X}}_{t}$). Alternatively small systematic errors on determining the solution uptake would reflect in relatively large changes in $\tilde{X}$ and ${\tilde{c}}_{\mathrm{calc}}$. Fig. 3 clearly shows that the coion exclusion in the conditions adopted in the present work is close to the ideal case with the electrical double layers totally overlapping (UP model), while for thin electrical double layers compared to the pore radius (SC model) highly overestimate ${\tilde{c}}_{\mathrm{calc}}$.

### 1.5 Streaming potential and figure-of-merit

Onsager proposed, based on the fundamental theorem of non-equilibrium thermodynamics, that, for sufficiently slow processes near equilibrium, the fluxes can be expressed as a linear combination of all the conjugated and non-conjugated driving forces [10], [11]. Using this framework the isothermal transport of an ideal electrolyte across a membrane can be described, in dimensionless formulation, as:

(6) $$u=L_{11}\left(-\frac{\partial p_{t}}{\partial x}\right)\;+\;L_{12}\left(-\frac{\partial \mu }{\partial x}\right)\;+\;L_{13}\left(-\frac{\partial \phi }{\partial x}\right)$$

(7) $$j_{\mathrm{ions}}=j_{+}+j_{-}=L_{21}\left(-\frac{\partial p_{t}}{\partial x}\right)\;+\;L_{22}\left(-\frac{\partial \mu }{\partial x}\right)\;+\;L_{23}\left(-\frac{\partial \phi }{\partial x}\right)$$

(8) $$j_{\mathrm{ch}}=j_{+}-j_{-}{=L}_{31}\left(-\frac{\partial p_{t}}{\partial x}\right)\;+\;L_{32}\left(-\frac{\partial \mu }{\partial x}\right)\;+\;L_{33}\left(-\frac{\partial \phi }{\partial x}\right)$$

in which: u, $j_{\mathrm{ions}}$ and $j_{\mathrm{ch}}$ represent the (dimensionless) volumetric solution, ion and electrical current flux, respectively; while the (dimensionless) driving forces: $\frac{\partial p_{t}}{\partial x}$, $\frac{\partial \mu }{\partial x}$ and $\frac{\partial \phi }{\partial x}$ are the total pressure, chemical potential and electrical potential gradient, respectively. Finally $j_{+}$ and $j_{-}$ represent the (dimensionless) flux of positive and negative ions, respectively. In general the cross-coefficients $L_{\mathrm{ij}}$ are concentration dependent and proven to be Onsager symmetric, hence $L_{\mathrm{ij}}=L_{\mathrm{ji}}$. In the absence of a concentration difference, i.e. $\frac{\partial \mu }{\partial x}$=0 and $L_{\mathrm{ij}}$=const, the system can be greatly simplified and in the integral form it can be rewritten as:

(9) $$J_{v}=L_{11}\left(-\frac{\Delta p_{t}}{\Delta x}\right)+L_{13}\left(-\frac{\Delta \phi }{\Delta x}\right)$$

(10) $$I{=L}_{31}\left(-\frac{\Delta p_{t}}{\Delta x}\right)+L_{33}\left(-\frac{\Delta \phi }{\Delta x}\right)$$

where *J*_v_ and *I* are the (dimensionless) volumetric solution flux and current density, respectively.

From Eq. (9) and Eq. (10) the most accessible experimental transport coefficients are the hydraulic permeability ($\kappa_{H}$), streaming potential coefficient (υ) and membrane conductivity (σ):

(11) $$\kappa_{H}=-J_{v}{\frac{∆x}{∆p^{h}}|}_{I_{m}=0}=K_{11}\left(1-\frac{{K_{13}}^{2}}{K_{11}K_{33}}\right)\left[m^{2}Pa^{-1}s^{-1}\right]$$

(12) $$\upsilon ={\frac{∆\varphi }{∆p^{h}}|}_{I_{m}=0}=-\frac{K_{13}}{K_{33}}\;[\mathrm{V P}a^{-1}]$$

(13) $$\sigma ={-I_{m}{\frac{∆x}{\varphi }|}_{∆p^{h}=0}=K}_{33}\;\left[Sm^{-1}\right]$$

in which the (dimensional) cross-coefficients *K*_*ij*_ can be calculated from the dimensionless counterparts as:

(14) $$K_{11}=L_{11}\frac{D}{c_{\mathrm{ref}}R_{G}T}\left[m^{2}Pa^{-1}s^{-1}\right]$$

(15) $$K_{13}=\frac{L_{13}D}{\Phi_{B}}\;[m^{2}V^{-1}s^{-1}]$$

(16) $$K_{33}=L_{33}\frac{\mathrm{FD}c_{\mathrm{ref}}}{\Phi_{B}}\;\left[Sm^{-1}\right]$$

where ${\tilde{c}}_{\mathrm{ref}}$ is the reference concentration (here 1 mol m^-3^ as in ref. [4]) and *D* is the average ion diffusion coefficient described as $D=\sqrt{D_{+}D_{-}}$ where $D_{-}$ and $D_{+}$ are the anion and cation diffusion coefficients, respectively.[7] For the calculation reported in this article the diffusion coefficients for the cation (Li^+^) and anion ($I_{3}^{-}$) were $D_{{\mathrm{Li}}^{+}}$ = 1.03·10^-9^ m^2^ s^-1^ [12] and $D_{I_{3}^{-}}$ = 1.10·10^-9^ m^2^ s^-1^, respectively.

**Fig. 4:**
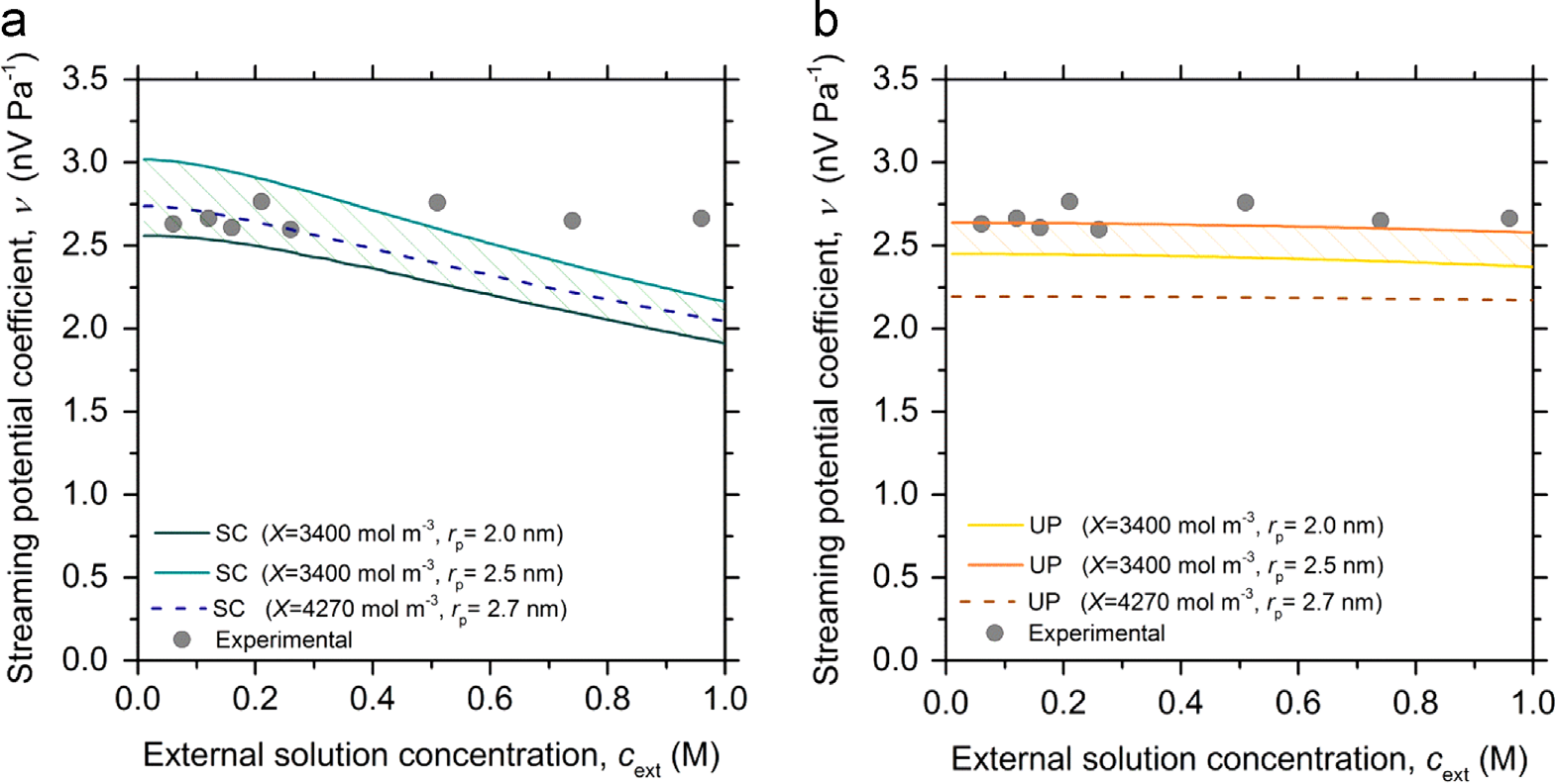
Streaming potential coefficient (υ) calculated from the phenomenological transport coefficients for different immobile charge densities ($\tilde{X}$) and pore radii ($r_{p}$). The phenomenological transport coefficients were determined using two different models; a) the Space charge (SC) and b) Uniform potential (UP), and plotted as function of the external LiI/I_2_ concentration $(c_{\mathrm{ext}})$. The experimentally determined streaming potential coefficients are represented by the solid symbols.

υ determined based on the SC and UP models are shown in Fig. 4a and b, respectively. It can be seen that υ for the SC model at a fixed geometry and immobile charge density, decreases with increasing external solution concentration while υ for the UP model is more or less concentration independent. The experimentally determined streaming potential coefficients are similar to the calculated ones from the UP model with a $\tilde{X}$=3400 mol m^-3^ and a pore radius of 2.5 nm.

In the data reported in Fig. 4 it has been assumed that the equilibrium LiI+I_2_⇌LiI_3_ was forced completely towards the triiodide for all used LiI/I_2_ solutions and hence the triiodide diffusion coefficient $D_{-}=D_{{I_{3}}^{-}}$ was used in the calculations. A sensitivity analysis of the influence of the diffusion coefficient on the calculated υ was performed by changing the anion diffusion coefficient to the iodide $D_{I^{-}}$ = 2.00·10^-9^ m^2^ s^-1^ [13]. $D_{I^{-}}$ is concentration dependent [13] and the highest value (i.e. at infinite dilution) was chosen to obtain the largest deviation from$D_{I_{3}^{-}}$. The percentage deviation between ν calculated with the two different anion diffusion coefficients were in most cases below 2% and increased at the most up to ~ 5% (for UP) and ~ 8% (for SC) for the most concentrated LiI/I_2_ solution.

The experimentally accessible transport properties described in Eqs. (11), (12), (13) are used to determine the Figure of merit (β) (Eq. (17)) according to the formulation given by Bentien et al. [14], which was derived using the framework proposed by Osterle and co-workers [2], [3], for the electrokinetic energy conversion to calculate maximum conversion efficiency ($\eta_{\max})$:

(17) $$\beta =\frac{{K_{13}}^{2}}{K_{11}K_{33}\;-\;{K_{13}}^{2}}=\frac{\sigma \cdot \upsilon^{2}}{\kappa_{H}}$$

**Fig. 5:**
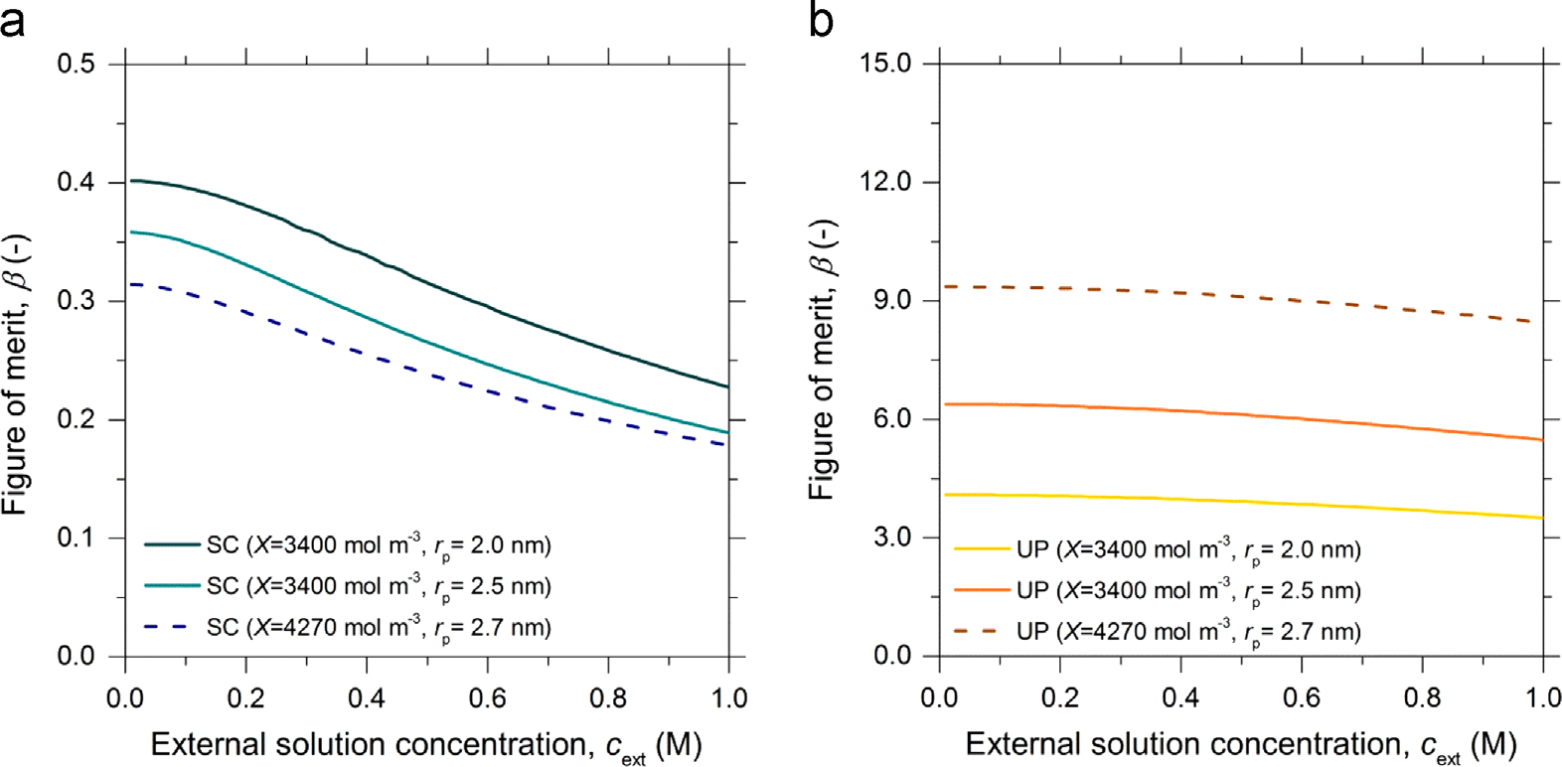
Figure of merit (β) shown as function of the external LiI/I_2_ solution concentration $(c_{\mathrm{ext}})$ for different immobile charge densities ($\tilde{X}$) and pore radii ($r_{p}$). β was calculated from the phenomenological transport coefficients derived considering the two different models; a) Space charge (SC) and b) Uniform potential (UP).

In Fig. 5 the β calculated using both the SC and UP models are shown for different $\tilde{X}$s and pore radii.

In the present work the UP model gives a better description of the experimentally determined $\tilde{c}$ and υ with respect to the SC model. It is important to recall that the quantitative estimation of β calculated from the UP model will in most cases of interest (pore radii > 0.5 nm) highly overestimate β [7].

## 2 Experimental design, materials and methods

### 2.1 Cell design

**Fig. 6:**
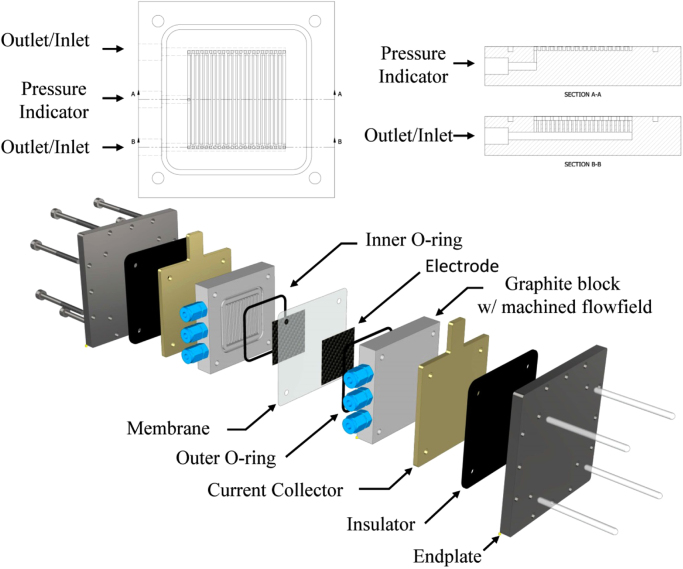
Exploded view of the flow cell and cross sections of the inlet/outlet and pressure indicator ports.

The flow cell described here was purposely designed and produced. The geometrical active area of the mounted membrane was 25 cm^2^. Fig. 6 depicts the exploded view of the flow cell and the graphite (Royal Elite New Energy Science & Technology, China) interdigitated flow field blocks where the ports for inlet, outlet and in-pattern pressure monitoring are shown. The inlet and outlet ports were 1/4 in. NPT threaded and coupled with straight 1/4 in. NPT to 1/4 in. compression fittings (Swagelok, PTFE). The ports for the pressure indicators were 1/8 in. threaded and 1/8 in. NPT to 1/8 in. compression fitting (Swagelok, PTFE) was used for the coupling with the instruments. The larger dimension for the fluid inlet and outlet was chosen to decrease the pressure drops in the external hydraulic circuit. Each cell (high pressure side and low pressure side) consisted of a stainless steel endplate, an insulator, a copper current collector, and a graphite block with machined flow field. The hydraulic sealing was ensured using an outer O-ring and an inner O-ring (M Seals, Viton) on the high and low pressure side, respectively. The membrane acted as an electrical insulator between the two electrodes. Two carbon paper sheets (FuelCellStore: Toray Carbon Paper, thickness: 190 µm) were placed in each graphite block between the flow field and the membrane. The flow field was lowered 0.3 mm with respect to the graphite block surface to ensure adequate space to host two carbon paper sheets (final compression of the carbon paper ~ 26% with an estimated porosity under compression ~ 74% [15], [16]). The interdigitated flow field, seen in Fig. 6, consisted of a single wall separating inlet and outlet streams forming 12 “dead-end” channels. This design forced the solution over the wall, through the porous carbon paper electrode, and close to the membrane where the electrokinetic phenomena took place.

### 2.2 Electrochemical impedance spectroscopy

For the Electrochemical Impedance Spectroscopy (EIS) analysis the used equivalent circuit and a representative spectrum can be seen in ref. [1] Figs. 1d and e, respectively. The equation of the equivalent circuit is:

(18) $$Z=R_{\mathrm{mem}}\;+\;\frac{1}{{\left(Y{\left(i\omega \right)}^{n}\right)}_{C}\;+\;{R_{C}}^{-1}}\;+\;\frac{1}{{\left(Y{\left(i\omega \right)}^{n}\right)}_{A}\;+\;{R_{A}}^{-1}}\;+\;\frac{1}{{\left(Y{\left(i\omega \right)}^{n}\right)}_{D}\;+\;{R_{D}}^{-1}}$$

Due to the imperfection of the capacitive elements in the flow cell, constant phase elements (CPE) have been used in the model. The exponent *n* measures how far the element (with C, A, and D representing the anode, cathode and diffusion limited element, respectively) is from a perfect capacitor (i.e. *n* = 1) or a resistor (*n* = 0). For this analysis *n* was allowed to vary between 0.7 and 1 [17]. In Table 2 all model parameters for the fits are given.

**Table 2:** Model parameters used to fit the experimental data regarding the flow cell resistance elements (*R*_*i*_), capacitance elements (*C*_*i*_) and constant phase element factor (0.7 ≤ *n*_*i*_ ≥ 1.0). These parameters describe an imperfect capacitor for membrane, anode, cathode, and diffusion limited parts of the flow cell electrical circuit with *i* = “mem”, “A”, “C”, and “D”, respectively. Parameters are shown for varying external LiI/I_2_ solution concentration (*c*_ext_) and for varying circulation flow rate (*q*_circ_).

**Varying external concentration at fixed*****q***_**circ**_**= 2.9 mL s**^**-1**^										
*c*_ext_ (M)	*R*_mem_ (Ω)	*R*_A_ (Ω)	*R*_C_ (Ω)	*R*_D_ (Ω)	*C*_A_ (F)	*C*_C_ (F)	*C*_D_ (F)	*n*_A_ (-)	*n*_C_ (-)	*n*_D_ (-)
0.06	0.057	0.281	0.128	0.107	2.72	0.60	460	0.7	0.7	0.840
0.12	0.055	0.163	0.102	0.046	5.00	0.87	1509	0.7	0.7	0.925
0.16	0.054	0.121	0.092	0.036	6.54	0.93	2255	0.7	0.7	0.927
0.21	0.053	0.099	0.076	0.018	7.79	1.22	4400	0.7	0.7	1
0.26	0.050	0.086	0.063	0.022	8.00	1.28	6820	0.7	0.7	0.979
0.51	0.043	0.042	0.032	0.006	8.48	3.77	10,181	0.844	0.736	0.7
0.74	0.041	0.029	0.036	0	8.19	7.03	–	0.729	0.7	–
0.96	0.043	0.029	0.029	0	8.50	41.00	1	0.7	0.7	1

**Varying external*****q***_**circ**_**at fixed*****c***_**ext**_**= 0.26 M LiI/I**_**2**_										

*q*_circ_ (mL s^-1^)	*R*_mem_ (Ω)	*R*_A_ (Ω)	*R*_C_ (Ω)	*R*_D_ (Ω)	*C*_A_ (F)	*C*_C_ (F)	*C*_D_ (F)	*n*_A_ (-)	*n*_C_ (-)	*n*_D_ (-)
0	0.064	0.083	0.047	0.614	37.93	3.15	449	0.7	0.7	1
0.2	0.063	0.037	0.046	0.016	37.90	3.04	110	0.7	0.7	1
0.7	0.061	0.039	0.040	0.020	21.98	3.65	1662	0.7	0.7	1
1.3	0.059	0.035	0.031	0.013	12.81	4.25	2062	0.7	0.7	1
2.1	0.056	0.029	0.029	0	7.00	3.01	–	0.7	0.7	–
2.9	0.056	0.027	0.027	0	3.20	5.02	–	0.7	0.7	–
3.2	0.057	0.027	0.027	0	3.00	4.99	–	0.7	0.7	–

### 2.3 Solution uptake and internal solution concentration

Nafion 117 samples in the H^+^ form were dried at 50 °C and the dry masses were measured. Afterwards the samples were equilibrated overnight in LiI/I_2_ solutions with concentrations in the range 0.06 to 0.96 M. Before measuring the wet membrane masses the membranes were carefully blotted with tissue paper to remove excess solution from the surfaces. The solution uptake (*s*) was determined for all LiI/I_2_ concentrations as:

(19) $$s=\frac{m_{\mathrm{wet}}-m_{\mathrm{dry}}}{m_{\mathrm{dry}}}\left[g_{\mathrm{sol}}g_{\mathrm{dry pol}}^{-1}\right]$$

where $m_{\mathrm{wet}}$ and $m_{\mathrm{dry}}$ are the wet and dry membrane masses, respectively. The average solution uptake for the LiI/I_2_ solutions in Nafion 117 was 21±2% (additional details can be seen in Table 1). No evident decrease in the solution uptake was observed due to membrane osmotic dehydration at elevated concentrations.

After the wet mass measurements the membranes were transferred to known volumes (*V*_sol_=20 mL) of milli-Q water (~18 MΩ cm) for 20 min to elute excess amounts of LiI/I_2_ according to the method described in ref. [18]. The elution was repeated to ensure complete LiI/I_2_ removal. The conductivity of the two solutions was measured using a conductivity probe (eDAQ, platinum plate electrodes, cell constant *k* = 10 cm^-1^). The solution concentration was afterwards calculated from the measured conductivity by means of a calibration curve (range 0.012 to 1.6 mM). The conductivity of the second solution was for all measurements equal to that of pure milli-Q water and was therefore not included in the determination of the eluted solution concentration ($c_{\mathrm{el}}$). $c_{\mathrm{el}}$ was used together with *s* to determine the concentration of sorbed LiI/I_2_ inside the membrane (${\tilde{c}}_{\exp}$). The internal solution concentration (henceforward the tilde symbol refers to properties inside the membrane) was calculated as:

(20) $${\tilde{c}}_{\exp}=c_{\mathrm{el}}V_{\mathrm{sol}}\frac{\tilde{\rho }}{s\;m_{\mathrm{dry}}}[\mathrm{mol}\;m^{-3}]$$

where $\tilde{\rho }$ represents the solution density inside the membrane which was approximated to the density of pure water ($\tilde{\rho }{}_{\sim }\rho_{w}$). The determined *s* and ${\tilde{c}}_{\exp}$ are shown in Table 1.

### 2.4 Ion exchange capacity – titration method

A titration method similar to the one described by Erbil and Baysal [19] was adopted for the determination of the effective ion exchange capacity (*iec*) at several LiI/I_2_ concentrations. The membrane samples were first equilibrated in the LiI/I_2_ solutions for 24 h. The membranes were then eluted in 2 × 20 mL milli-Q water for 20 min to remove excess amounts of LiI/I_2_. Afterwards the samples were transferred to 2 × 10 mL of 0.050 M HCl for one day per time. The solutions were combined (*V*_tot_ = 20 ml) and known sample volumes (*V*_sample_) were titrated with 0.050 M NaOH (*c*_NaOH_) (Metrohm Autotitrator (916Ti-Touch)). The original 0.050 M HCl solution was titrated as well. The difference in titrant volume at the equivalence point between the pure HCl solution (*V*_titr,HCl_) and the sample solution (*V*_titr,mem_) represents the amount of protons exchanged with the membrane. The *iec* was determined for the most concentrated LiI/I_2_ solutions (0.26, 0.51, 0.74 and 0.96 M) as:

(21) $$\mathrm{iec}=\frac{\left(V_{\mathrm{titr},\mathrm{HCl}}\;-\;V_{\mathrm{titr},\mathrm{mem}}\right)\cdot c_{\mathrm{NaOH}}}{m_{\mathrm{dry}}}\cdot \frac{V_{\mathrm{tot}}}{V_{\mathrm{sample}}}\left[\mathrm{meq}\;g_{\mathrm{pol}}^{-1}\right]$$

An average *iec* of 0.80±0.02 $\mathrm{meq}g_{\mathrm{pol}}^{-1}$ was found which is slightly lower than the value reported from the manufacturer ~ 0.91 meq $g_{\mathrm{pol}}^{-1}$ [20], [21] (see Table 2). From *iec* and *s* the immobile charge density per unit volume of solution inside the membrane $\left(\tilde{X}\right)$ can be determined as:

(22) $$\tilde{X}=\mathrm{iec}\frac{\tilde{\rho }}{s}\left[\mathrm{mol}\;m^{-3}\right]$$

where also in this case $\tilde{\rho }\sim \rho_{w}$. The *iec* from the titration experiments were used to determine the immobile charge densities (${\tilde{X}}_{t}$) (see Table 1) with an average value of ~3400 mol m^-3^ while an immobile charge density of 4270 mol m^-3^ (${\tilde{X}}_{s}$) was determined using *iec=*0.91 meq $g_{\mathrm{pol}}^{-1}$.
