# Supplementary material for: Data on flow cell optimization for membrane-based electrokinetic energy conversion
Source: Data Brief. 2017 Sep 1;15:1–11. doi: 10.1016/j.dib.2017.08.036 (PMC5712061; doi:10.1016/j.dib.2017.08.036)
Supplement: Supplementary file 1 — Supplementary material [file mmc1.pdf]

To the Editor

Conflict of Interest statement

To the Editor

I hereby confirm on behalf of all authors that we do not have any conflict of interests.

Kind regards

Anders Bentien
Associate Professor

Chemistry & Biotechnology
Department of Engineering

Anders Bentien

Associate Professor

Date: 16 May 2017

Direct Tel.: +45 3036 9515
E-mail: bentien@eng.au.dk

Web: www.eng.au.dk
